# Rates and Patterns of Australian Emergency Department Presentations of People Who Use Stimulants: A Systematic Literature Review

**DOI:** 10.7759/cureus.30429

**Published:** 2022-10-18

**Authors:** Peter T Redona, Cindy Woods, Debra Jackson, Kim Usher

**Affiliations:** 1 Nursing, University of New England, Armidale, AUS; 2 Substance Use and Addiction, Health Research Institute, University of Canberra, Canberra, AUS; 3 Faculty of Health and Medicine, University of Sydney, Sydney, AUS; 4 Faculty of Health and Medicine, University of New England, Armidale, AUS

**Keywords:** methamphetamines, cocaine, emergency departments, amphetamines, stimulants use

## Abstract

The use of illicit stimulants continues to pose a significant challenge to different health sectors. In Australia, four particular stimulants, namely amphetamines and their derivatives, methamphetamine, ecstasy or 3,4-methylenedioxy-methamphetamine (MDMA), and cocaine cause a significant challenge to EDs as managing patients who use stimulants can be labor and resource intensive. While Australian data are available for stimulant-related ambulance attendances and hospitalizations, little is known about ED presentations of people who use stimulants. The aim of this paper is to systematically review the available literature related to the rates and patterns of ED presentations of people who use stimulants in Australia. A search was conducted on EBSCOhost, CINAHL Complete, and PubMed databases, as well as Google Scholar. Search terms consisted of combinations of the following terms: 1) stimulant AND ED AND Australia; 2) stimulants AND emergency presentations OR accident and emergency AND Australia, 3) amphetamine OR methamphetamine OR ecstasy OR cocaine AND ED AND Australia. Articles that met the inclusion criteria were included in the review and subjected to a quality appraisal. Data were extracted from the selected papers, including patient demographics, presentation rates, type of stimulant, reasons for presentations, police or ambulance service involvement, comorbidities, mental health issues, triage codes, admissions, and separations. The results of the review are reported using the Preferred Reporting Items for Systematic Reviews and Meta-Analysis (PRISMA) guidelines.

Studies were eligible if they were English-language peer-reviewed articles published between January 2011 and December 2021 and if they included data on Australian ED presentations of people who use non-prescription illicit stimulants. Studies were excluded if they did not include stimulant-related ED presentations or focused on ED presentations related to prescription stimulants, including Ritalin and Adderall, non-stimulant drugs, or caffeine for attention deficit disorder (ADD) or attention-deficit hyperactivity disorder (ADHD). The selected articles were appraised for quality, rigor, and risk of bias by two authors. The studies were assessed using the Newcastle Ottawa Scale (NOS) for cross-sectional, cohort, and case-control studies depending on the methodology identified in the study. A total of 19 articles were included in this study. Males represented 53 to 85% of ED presentations of people who use stimulants with an age range of 0 to 65 and are more likely to be transported by police or ambulance. People who use stimulants presented to EDs with varying psychological and behavioral concerns such as psychosis, self-harm, suicidal ideations, hallucinations, agitations, and aggressiveness, as well as medical conditions, including heart palpitations, nausea and vomiting, and significant physical injuries.

## Introduction and background

The WHO (2018) reported that the use of stimulants is a rapidly growing global issue. While the geographical spread of this epidemic is widening, in many countries, awareness of the problem remains limited [1]. The Institute for Health Metrics and Evaluation or IHME reported that in 2019, amphetamine use resulted in 1.40 million global disability-adjusted life years (DALYs) and was responsible for 0.1% of total global DALYs (Global Burden of Disease [GBD] for 2016) [2]. Furthermore, according to a systematic analysis of the GBD for 2015, the death rates for all ages and genders for amphetamine and cocaine use disorders have risen significantly compared to the rates in 2005 [2]. The death rate ascribed to amphetamines and cocaine use in 2005 was 7.3 for amphetamines and 7.4 (per 100,000 deaths) for cocaine, compared with the death rate in 2015 of 12.8 and 11.1 (per 100,000 deaths), respectively [2].

In the United Kingdom, according to the European Monitoring Centre for Drugs and Drug Addiction (EMCDDA) (2017) [3], after cannabis, stimulants like cocaine, ecstasy, amphetamines, and amphetamines derivatives are the most commonly used illicit substances in the country. In terms of gender breakdown per stimulant type, a larger proportion of men (5.8%) used cocaine compared with women (2.2%). A similar pattern was seen in amphetamine use, with 1.3% of males and 0.6% of females reporting to have used the drug. The same trend can be seen in ecstasy, with more men admitting having used ecstasy (4.6%) than women (1.7%) [3].

According to the most recent report of the Australian Institute of Health and Welfare (AIHW) [4], in 2019, the recent consumption of amphetamine-type stimulants (ATS) decreased from 3.4% in 2001 to 1.3% among people aged 14 and over. In contrast, the use of cocaine rose significantly from 2.5% in 2016 to 4.2% across all age groups. From 2019 to 2020, amphetamines were the most common principal drug of concern, amounting to 28% of closed treatment episodes. However, in the same year, Australia had the fourth-highest average total consumption of stimulants compared to other countries in Europe, Oceania, South Africa, and North America [4]. However, Australia had the highest levels of amphetamine dependence worldwide, according to the Global Burden of Diseases, Injuries, and Risk Factors study [5]. Methamphetamine-related deaths in Australia have also increased as the mortality rate between 2009 and 2015 shows the mortality rate to have doubled in the seven years interval [5]. Furthermore, the AIHW reported that the use of methamphetamines is disproportionately higher for specific population groups, such as Aboriginal and Torres Strait Islander people, who are twice as likely to report the recent use of methamphetamine than non-Indigenous Australians [4]. Similarly, people with mental health conditions were twice as likely to report recent use of amphetamine or methamphetamine than those without mental health conditions [4].

Rationale

The escalating use of stimulants in the Australian community warrants the study of the rates and patterns of stimulant-related presentations in Australian EDs to provide information that could inform government and health organization policy and practice. This can be relevant to assist hospital management and emergency organizations to be more prepared to address this national problem. According to a recent literature review, there is limited literature that describes the pattern and scale of methamphetamine-related presentations in EDs globally [6]. Most studies on ED presentations of patients who use stimulants used data from a limited number of hospitals in just one or two states. Additionally, most of these studies only detailed ED presentations related to a single type of stimulant. The authors concluded that there are difficulties in capturing the exact figures for ED presentations of people who use methamphetamines due to difficulties in screening and reliance on self-report or subjective clinician diagnosis [6]. As stimulant use causes physical symptoms, such as increased heart rate and blood pressure, loss of muscle control, and mental health conditions, including paranoia, hallucinations, and sometimes psychotic and violent behaviors [7], presentations to EDs resulting from these side effects may be misinterpreted or wrongly reported. For example, presentations to EDs related to stimulant use may be classified as stimulant-related due to the specific presenting signs and symptoms, with the precipitating stimulant used not identified. Another difficulty is polysubstance use, in which stimulants may be used in conjunction with other illicit substances or alcohol. Therefore, acquiring the exact figure of ED presentations attributable to stimulants poses some challenges. This review was undertaken to ascertain current knowledge about rates and patterns of stimulant-related presentations to Australian EDs; thus, the review was limited to Australian studies.

## Review

Objective

To systematically review the available literature related to the rates and patterns of ED presentations of people who use stimulants in Australia.

Methods

**Figure 1:**
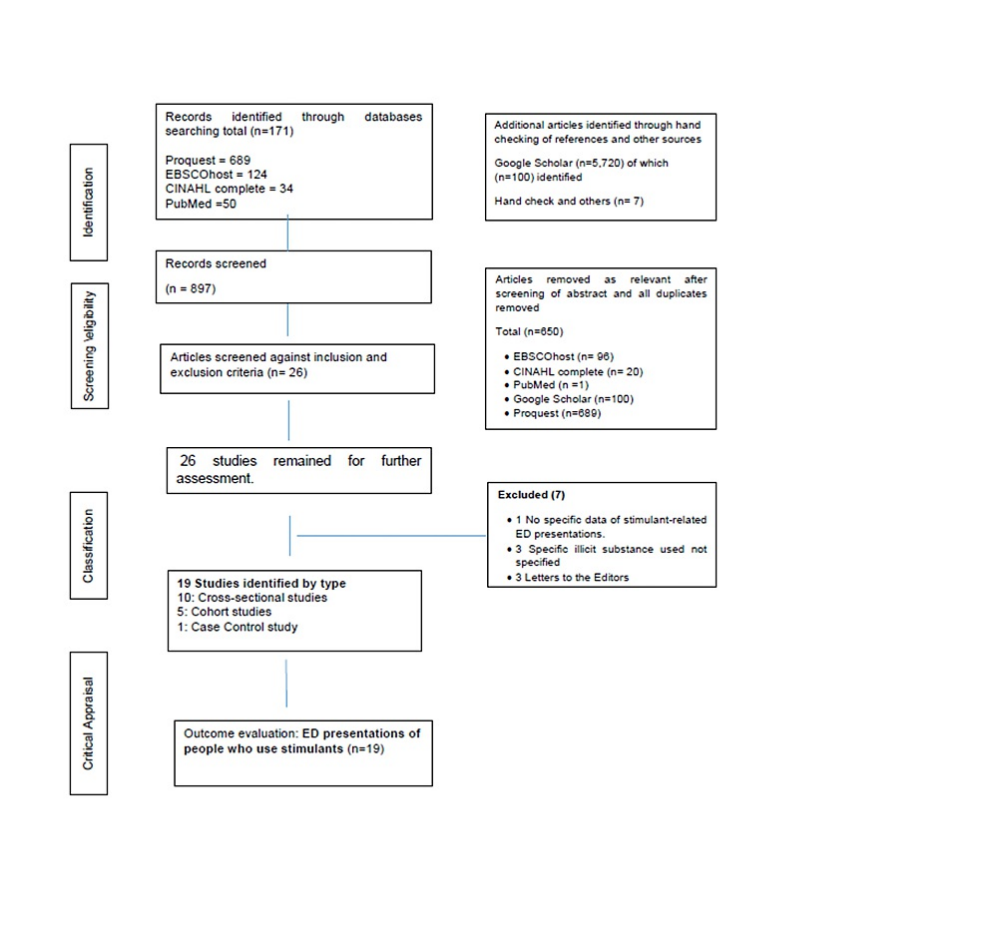
PRISMA flow diagram summarizing systematic search on the Australian ED presentations of people who use stimulants. PRISMA: Preferred Reporting Items for Systematic Reviews and Meta-Analysis.

A systematic literature review was conducted using literature published over 10 years, from 2011 to 2021. The 10-year timeframe was chosen to track changes in rates and patterns of ED presentations of people who use stimulants to better understand the recent impact on emergency services in Australia. Additionally, the increase in supply, popularity, and consumption, and media reporting of stimulant use has become more prominent in the last decade, resulting in clinicians becoming more aware of stimulants as a possible cause of intoxication, psychosis, violence, aggression, and other health-related consequences [7]. The results of the review are reported following the Preferred Reporting Items for Systematic Reviews and Meta-Analysis (PRISMA) guidelines (Figure 1).

Eligibility criteria

Studies were eligible if they were English-language peer-reviewed articles published between January 2011 and December 2021 and included data on Australian ED presentations of people who use non-prescription illicit stimulants. Studies were excluded if they did not include stimulant-related ED presentations or focused on ED presentations related to prescription stimulants including Ritalin and Adderall, non-stimulant drugs, or caffeine for attention deficit disorder (ADD) or attention-deficit hyperactivity disorder (ADHD).

Information sources

A search was conducted on ProQuest, EBSCOhost, CINAHL Complete, and PubMed databases as well as Google Scholar.

Search strategy

A specialist health science librarian was consulted to assist with the design of the search strategy. Search terms included combinations of the following terms: 1) stimulant AND ED AND Australia; 2) stimulants AND emergency presentations AND Australia; 3) amphetamine OR methamphetamine OR ecstasy OR cocaine AND ED AND Australia. Boolean limiters were used to narrow the search using the Boolean operator "NOT." For example, the operator NOT was combined with words and phrases such as ADHD, ADD, Ritalin, and caffeine. Using the listed search terms, the first 100 articles in Google Scholar were also scanned and reviewed for inclusion in the study.

Selection process

The first author scanned searched titles and abstracts for eligibility, noting reasons for ineligibility. Two authors (Peter Redona and Cindy Woods) then independently reviewed the selected full-text articles against the inclusion and exclusion criteria. Where concordance was not achieved following a discussion, the articles were referred to a third author to adjudicate. See Table 1 for inclusion and exclusion criteria.

**Table 1:** Inclusion and exclusion criteria.

Inclusion Criteria	Exclusion Criteria
-Peer-reviewed empirical research published from January 2011 to December 2021. -Articles that included data on ED presentation due to stimulant use, specifically amphetamine, speed methylamphetamines, cocaine, and ecstasy. -English language full-text articles.	-ED presentation related to prescription stimulants for attention deficit disorder (ADD) or attention-deficit hyperactivity disorder (ADHD), including Ritalin and Adderall. -Research related to ED presentations related to non-stimulant drug use. -Research related to caffeine as a stimulant. -Research that presents stimulant-related presentations to hospitals without presentation to EDs. -Journal articles published prior to January 2011

Data collection process

Data were extracted from quantitative studies to describe statistical information on stimulant-related ED presentations in Australian EDs. The data were extracted in numerical and textual format and then summarized in a table. The data extracted included comprehensive information about the population, setting, methodology, aim, findings or outcomes, and limitations of the studies. One author (Peter T. Redona) independently extracted data from the included studies, and the data were then reviewed by a second author (Cindy Woods). Any discrepancies were discussed and resolved or referred to a third author for resolution.

Data items

Outcomes included presentation rates, screening tools used, types of stimulants, the reason for presentation, comorbidities, types of hospital separation, and triage scale category using the Australian Triage Scale (ATS). The ATS is a clinical tool that defines the maximum waiting time for medical assessment and treatment [8]. The ATS consists of a five-point scale that prioritizes patients based on clinical urgency. For example, category one is the most urgent life-threatening category and requires immediate treatment; category two is defined as urgent and requires treatment within 10 minutes; category three is less urgent and requires treatment within 30 minutes; category four requires treatment within 60 minutes; and category five requires treatment within 120 minutes [8].

Risk of bias assessment

Selected articles were appraised for quality, rigor, and risk of bias by two authors. Peter T. Redona initially appraised the studies, and then Cindy Woods reviewed the studies, with any differences reviewed until an agreement was reached. The studies were assessed using the Newcastle Ottawa Scale (NOS) for cross-sectional, cohort, and case-control studies depending on the methodology identified in the study. The NOS is one of the most used scales for assessing quality and risk of bias in observational studies. Different versions can be used for cross-sectional, case and longitudinal, or prospective studies [9]. The NOS rates a study on three quality perspectives: selection of study groups, comparability of study groups based on the analysis or study design elements, and the outcome of interest or ascertainment of exposure for cohort or case-control studies. These parameters are divided across eight specific items and are scored from one point; however, comparability can be modified to cater to the particular topics of interest and can score up to three points [9]. The overall category, though not clearly stated in the NOS manual, is determined based on the total scores of the studies; 0 to 4= unsatisfactory; 5-7= satisfactory; 8 to 9= good [10].

Reporting bias assessment

The 19 studies included in this literature review fall into three different categories. Eleven studies were cross-sectional, seven were cohort and a single case-control study. Specific variations of the Newcastle Ottawa scale were applied in the quality assessment of the studies.

Results

The initial search identified 897 articles. After duplicates were removed, 650 articles remained. Titles and abstracts were screened, and 247 records were excluded based on exclusion criteria. The remaining full-text articles were screened for inclusion. Of the final 26 articles, three were removed as the stimulant/s that led to the admission were not specified, three were Letters to the Editors, and one article was excluded as no ED admission data were identified. Nineteen articles met the inclusion criteria and were included in the review.

Age and gender

In 14 of the selected studies, males represented the majority of ED presentations of patients who use stimulants, with admission percentages ranging from 53% [11] to 85% [12]. The age of the research population ranged from 0 years [13] to 65 years [14]. Ten of the studies identified the average age of their research population with a median age between 28 and 36 [11,15-23]. In the two studies that included ED presentations due to the use of methylenedioxymethamphetamine (MDMA) or ecstasy, most patients were in the 20-35 age group [15,24]. Only one of the studies involved pediatric presentations to EDs aged 0-19 years old [13].

Screening tools

Only one of the 19 studies used an assessment tool to determine cases of substance use-related ED presentations. Butler K et al. [11] used the Alcohol Smoking and Substance Involvement Screening Test or ASSIST. This tool screens for the use of different substance types, including alcohol, tobacco products, amphetamine and amphetamine-type stimulants (ATS), cannabis, cocaine, sedatives, hallucinogens, inhalants, opioids, and 'other' drugs. The tool used three different risk scores; lower, moderate or high [11]. The results of this tool, used to identify the type of drugs that patients used, were significantly comparable to other measures used to screen for substance use. Four studies [12,14,25,26] used urine analysis to determine the stimulant used by the patients presenting to ED. However, only one of the studies mentioned the use of blood analysis to determine the psychoactive substance used by the patient presenting to EDs [14]. Three studies used the patients' saliva to determine the presence of stimulants among the patients presenting to EDs [12,22,23].

Main stimulant used

All of the selected studies analyzed ED presentations that were related to non-prescription stimulants, namely, amphetamine-type stimulants, methamphetamine or ice, MDMA or ecstasy, and cocaine. Four of the studies [12,19,22,25] focused on methamphetamine-related ED presentations. In six of the studies [11,15-17,23,27], amphetamine and/or amphetamine-type stimulants were the main drug of concern. In contrast, Horyniak D et al. [24] focused only on ecstasy-related ED presentations. The rest of the studies examined ED presentations due to multiple substance use, including stimulants, alcohol, and other drugs.

Methods to identify the study population

Eighteen of 19 studies conducted audits of emergency and hospital medical records to determine the study population of stimulant-related ED presentation. Only one study used a screening tool to determine the research population [11]. In contrast, another study conducted an audit of patient information of participants recruited as part of a methamphetamine treatment evaluation study (MATES) to research stimulant-related ED presentations [19].

Two distinct classification methods were used to determine the diagnoses of patients included in the studies. The International Statistical Classification of Diseases and Related Health Problems, Tenth Revision, Australian Modification (ICD-10-AM) codes were used to determine the diagnoses of patients in six of the studies [13,15-17,21,28]. In comparison, four of the selected studies [11,14,19,22] used the Diagnostic and Statistical Manual of Mental Disorders, 4th Edition (DSM-IV) to determine the diagnosis of the mental illness of patients in their respective research populations. The other studies relied on triage notes and patients' self-reports to determine the diagnoses based on the type of stimulant used by the patient presenting to the ED.

Percentage of ED presentation of patients who use stimulants

Seven of the 19 studies compared the percentage of stimulant-related presentations to the number of ED presentations in the study period with varying results. Percentages ranged from 0.15% of the total ED presentations [27], 1.2% [16], 2%, [11], 2.4-3% [24] and 11.9% [21]. Di Rico R et al.'s study reported that 7% of total ED presentations were due to mental and behavioral disorders involving psychoactive substances [28]. Although McKetin R et al. did not specify a percentage, it was estimated that 29,700-151,800 additional ED presentations could be attributed to methamphetamine use [19]. McKenna B et al.'s study reported that among the cohort of patients with consecutive admissions into the inpatient unit, 30.6% either tested positive for methamphetamine or admitted usage through self-report [22]. Gerdtz M et al. reported that 23.8% of ED behavioral unit presentations involved the presence of amphetamine-type stimulants [23]. In the study that investigated workplace violence in the ED, stimulants were involved in at least 41.5% of cases, and the most common stimulant used was methamphetamine [29].

Reason for ED presentation and comorbidities

Stimulant-related psychosis was a significant cause of patients presenting to the EDs [14-16,18,28]. These researchers claim that people who use stimulants are more likely to develop psychosis than the general Australian population. Various comorbidities were also identified in the ED attendance due to stimulant use. Two studies [12,25] found that patient admissions to the ED in relation to stimulant use were also due to mental health concerns, including psychosis. Conditions that were seen in stimulant-related presentations include heart palpitations, psychosis, agitation, anxiety and behavioral concerns, nausea, and vomiting [15,16, 24]. Hiscock H et al.'s study that focused on pediatric ED presentations due to stimulant use identified intentional self-harm and psychoactive substance use were the most frequent reasons for mental health presentations [13]. McKenna B et al. highlighted that among ED presentations involving methamphetamine use, consumers were more likely to have a mental illness as their primary diagnosis [22].

Mental health concerns were frequently seen in patients that presented to the EDs due to stimulant use; one study reported major depression in 44% of methamphetamine-related ED presentations, 37% for panic disorders, and 17% for schizophrenia [18]. According to three studies, psychosis was more likely to be present in patients with a stimulant-related ED presentation when compared to other mental illnesses [16,17,18]. Physical injuries, aggression, and suicide risks were identified as reasons for the ED presentation in five studies [12,14,22,24,27]. Unadkat A et al. reported that 66% of people presenting to the EDs who have used amphetamine were aggressive towards staff, while 50% were aggressive towards other patients [12]. Furthermore, in one study, 69% of patients required physical restraint, while 62% needed mechanical restraints [12]. Mechanical restraint refers to the use of devices (including belts, harnesses, manacles, sheets, and straps) to a person's body to restrict movement. In contrast, physical restraint is the application of hands-on immobilization by healthcare professionals or the use of physical restriction to prevent patients from harm to themselves or others or to ensure that essential medical treatment can be provided [30]. Chemical restraints, i.e., the use of tranquilizers and sedatives, were also discussed in two of the studies. One study used tranquilizers and oral sedation in 15.9% and 14% of patients, respectively [27]. In contrast, another reported that oral sedation was administered in 61% of the stimulant-related presentations [25]. In addition, a study identified that amphetamine-related presentations are quite distinct from other drug-related ED presentations, reporting that patients are more likely to present with psychiatric or behavioral concerns or altered levels of consciousness [24]. Lastly, a study reported that 10.8% of the presentations due to methamphetamine use reported the use of restrictive interventions [22].

Eight studies identified ambulance attendance in ED presentations of patients who used stimulants. A total of 73.2% of patients identified to be positive for stimulant use arrived via an ambulance [23], while another study reported that 62% of ecstasy-related ED presentations required ambulance services [24]. Five other studies reported various percentages of stimulant-related ED presentation requiring ambulance services; [14] 11.9%, [19] 27%, [27] 37%, [25] 41%, and [15] 52%. Furthermore, it was reported in one study that all methamphetamine-related ED attendances (n=637) were transported to the ED via ambulance services [18].

Police transport to EDs and further police involvement were also reported in eight of the studies. According to McKenna B et al., 59.1% of ED presentations involving methamphetamine use were transported by and required the presence of police [22]. Another study reported that 47.6% of ED presentations related to stimulant use were transported and attended by the police [14]. Other studies also reported police transport and attendance in 38.9% of presentations [18], while Monahan C and Coleman M (2018) reported that 23% of the ED presentation also needed the presence of the police [26]. Police transport and attendance were reported in the following studies [15,23,25,27] at percentages of 21.6%, 15%, 11%, and 4%, respectively.

Emergency categories

Five studies reported the emergency triage code of stimulant-related presentations based on the Australasian Triage Scale (ATS). A total of 4% of stimulant-related ED presentations were triaged as Category 1, requiring immediate care [19]. However, the classification of the other stimulant-related presentations was not specified. One study reported stimulant-related presentations that were triaged as Category 3 or Urgent [15]. This triage category requires that the patients are treated or seen by the medical practitioner within 30 minutes [8]. A total of 68.8% of ED presentations for amphetamine-type stimulants were triaged as Category 3, 68.2% of presentations for ecstasy or MDMA, and 75% for cocaine-related ED presentation [15]. In addition, 3% of ecstasy-related ED presentations were triaged as Category 1 (a life-threatening emergency condition), whereas 4% of amphetamine-related ED presentations were triaged as Category 1 [24]. In a more recent study, it was reported that 13.4% of patient presentations involving the use of amphetamine-type stimulants needed immediate emergency treatment (Category 1), 11.3% needed to be assessed and treated within 10 minutes (Category 2), and 53% were triaged as Category 3 and required assessment within the next 30 minutes [23].

Admission requirements

Seven studies identified the need for either hospital admission or further healthcare services for their research population. A total of 39% of amphetamine-related ED presentations required hospital admission [16]. However, in one study, only 14% of methamphetamine users required hospitalization [24]. A total of 34.4% of amphetamine-type stimulant-related presentations required admission to a psychiatric hospital, while 3.2% needed a referral to drug and alcohol services [27]. A study reported that 21% of amphetamine-type stimulant-related patients required hospital admission, and 16% were primarily due to psychiatric disorders [26]. People with stimulant use disorders alone had higher rates of self-harm, infectious disease, and non-mental health admissions than those who use cannabis [17]. A total of 84% of methamphetamine-related ED presentations were managed solely within the ED, with a median length of stay of 14 hours [25]. However, the study also reported that 1.6% and 1.9% of patients were admitted to surgical and medical wards, respectively, and 2.8% were admitted to the ICU [25]. Among patients presenting due to methamphetamine use, 74.6% required involuntary admission [22]. Lastly, ED presentations involving amphetamine-type stimulants required either psychiatric or medical admissions at 9.3% and 1%, respectively [23].

Risk of bias

Based on the quality appraisal, total NOS scores ranged from 4 to 8 (maximum score of 10), suggesting that most studies were unsatisfactory, satisfactory, and good, with eight studies scoring 7-8 stars (good), eight studies scoring 5-6 (satisfactory), and three studies, scoring 0-4 (unsatisfactory). Most studies (17 out of 19) scored 3-4 in the selection category (maximum of six stars), with just two studies scoring two stars. This indicates that the study population was clearly representative of the required exposure or drug of interest. The scores for comparability (maximum of 2 stars) were variable, with 10 of the studies having no scores in this category. Six of the studies scored two stars in this category, and the rest of the studies scored just 1 star. This result is probably due to most of the studies being retrospective analyses of medical records limiting control of potential confounders. Lastly, for the outcomes assessment (maximum 3 stars), the studies scored significantly high in this category; three studies scored 3 stars, 12 scored 2 stars, and four scored 1 star. This category reflects confirmation of outcome by reference to secure medical records and adequacy of follow-up of cohorts. Table 2 presents the result of the quality assessment of the studies using the NOS.

**Table 2:** Quality assessment of the studies using the Newcastle Ottawa Assessment scale.

Author (Year)	Study Design	Selection Maximum 4 to 6 stars	Comparability Maximum 2 stars	Outcome Maximum 3 stars	Total 10 stars
Arunogiri et al. (2016), [18]	Cross-sectional	3	Not applicable	3	6: Satisfactory
Butler et al. (2016), [11]	Case-control	2	2	2	6: Satisfactory
Chivaurah et al. (2019), [27]	Cross-sectional	3	Not applicable	1	4: Unsatisfactory
Di Rico et al (2018), [28]	Cohort	2	Not applicable	2	4: Unsatisfactory
Fatovich et al. (2012), [16]	Cohort	3	1	3	7: Good
Gerdtz et al. (2020), [23]	Cross-sectional	3	Not applicable	2	5: Satisfactory
Hiscock et al. (2018), [13]	Cross-sectional	3	Not applicable	2	5: Satisfactory
Horyniak et al. (2014), [24]	Cross-sectional	4	Not applicable	2	7: Good
Indig et al. (2010), [15]	Cross-sectional	3	2	2	8: Good
Isoardi et al. (2019), [25]	Cross-sectional	3	Not applicable	2	5: Satisfactory
Latt et al. (2011), [14]	Cross-sectional	3	Not applicable	2	6: Satisfactory
McKenna et al. (2017), [22]	Cohort	4	2	2	6: Good
Mcketin et al. (2017), [19]	Cohort	4	1	2	7: Good
Monahan and Coleman (2018), [26]	Cross-sectional	3	Not applicable	2	5: Satisfactory
Nikathil et al. (2018), [29]	Cohort	3	2	1	6: Satisfactory
Nambiar et al. (2011), [21]	Cohort	3	1	1	6: Good
Sara et al. (2014), [17]	Cohort	3	2	2	8: Good
Sara et al. (2017), [20]	Cross-sectional	3	2	3	8: Good
Unadkat et al. (2019), [12]	Cross-sectional	3	Not applicable	1	4: Unsatisfactory

Discussion

The ED presentations of people who use stimulants are frequently due to mental health concerns, such as agitation, psychosis, and aggression, and can be accompanied by other physical comorbidities. The results of this review indicate that young males aged 20-35 are more likely to present to the EDs with stimulant-related issues. They are also frequently transported to the EDs by police or ambulance or with both in attendance. A large number of polydrug-related presentations were also reported [19]. These results indicate that patients presenting to the EDs due to stimulant use are more likely to require additional emergency services resources linked to aggressive and abusive behaviors associated with stimulant use, posing safety and security issues for ED frontline workers. These findings indicate that stimulant use can cause a burden on police, ambulance, and hospital resources.

The gender imbalance in drug-related ED presentations is not new. A recent literature review involving methamphetamine-related ED presentations in different countries [6] reported that in most of the studies, a higher percentage of males presented to the EDs compared with females, with the percentage of males ranging from 61% to 85% of total methamphetamine-related ED presentations. Several international studies present similar data in terms of the gender of stimulant users. A study conducted in the USA [31] found that males represented the majority of the research participants who used amphetamines (51.4%). In a study about the use of ecstasy that was conducted in Israel, the research participants were also dominated by males (57%) [32].

Similarly, the European drug report in the United Kingdom published by the EMCDDA revealed that more men had used cocaine, ecstasy, and amphetamines [3]. This is also reflected in the 2017 World Drug report published by United Nations on Drugs and Crime (UNODC), which reported that, in general, twice as many men suffer from drug use worldwide compared to women [33]. Australian data also shows that more males than females self-report using stimulants [4].

It is concerning that stimulant use was reported in a study involving pediatric patients. In a study involving patients aged 0 to 19 years old with mental health problems related to psychoactive substance use, 22.3% were identified as the second largest category of ED presentations in Victorian public hospitals, second only to intentional self-harm (22.5%) [13]. However, it is essential to note that the study did not report which age group presented to the ED due to the use of psychoactive substances. A retrospective audit of data obtained from the local Emergency Department Information System was conducted for all mental health presentations to the Prince Charles Hospital Children's Emergency Department (TPCH-CED) Brisbane between January 1, 2013, and January 1, 2018 [34]. In the study, around 6.3% of pediatric patients presenting to TPCH-CED due to mental health issues had a history of substance use, and 3.3% were due to concomitant illicit drug intoxication [34]. The study did not identify the main drugs of concern for pediatric ED presentation. However, it does highlight that substance use can also be a cause of concern for ED personnel dealing with younger demographics, as the study reported that 95-98% of the pediatric ED presentations were from ages 12 to 16 years.

ED presentations due to the use of stimulants are also more likely to present with various comorbidities ranging from self-harming behaviors, depression, psychosis, and seizures as well as heart conditions, for instance, tachycardia, necessitating the need for immediate treatment [18] or sometimes even death due to cardiac arrest [25].

Patient presentations due to stimulant use ranged from 0.10% [15] to 3% [24], depending on the type of stimulant involved. Research involving stimulant-related presentations conducted in other countries presents similar scenarios to the findings of this study [3,31]. Although these figures are relatively small, ED presentations of people who use stimulants create a disproportionate use of emergency services and resources. These include the involvement of police and ambulance services, the use of restraints and a safe room to protect the patient from self-harm and other patients in the ED waiting room from potential violence and aggression, and mental health services [35]. Healthcare professionals that cared for patients presenting to the EDs due to stimulant use found the situation volatile and dynamic and that the staff could only manage the physical, mental, and behavioral issues of the patients by working together as a team, involving the services of health and social care professions [35].

In the 2017 European Drug Report, 65% of drug-related presentations to the EDs of 15 sentinel hospitals in nine European countries involved drug use, including heroin, cocaine, amphetamine, and MDMA [3]. In Spain, cocaine was involved in 50% of the total drug-related emergencies. At the same time, in Slovenia, there is an upward trend in drug-related ED presentations involving cocaine, amphetamines, and gamma hydroxybutyrate (GHB) [3]. Furthermore, in Europe, presentations related to cocaine use as a review of the latest national reports submitted to the EMCDDA by several European countries show that seven out of 10 reports point to an increase in cocaine-related emergencies compared to the previous year [36]. In addition, a new type of stimulant with the chemical name 4-flouroamphetamine is a new concern in sentinel regions in the Netherlands, with 272 emergency presentations in 2016 attributed to this novel nervous system stimulant [3]. The UNODC 2017 report also linked the effects of stimulant use to ED presentation, noting that around 16% of ED presentations globally can be attributed to cocaine toxicity [3].

Worldwide, amphetamines continue to be the second-most used stimulant, with the UNODC estimating that in 2015, around 35 million people used amphetamines in the previous year [33]. Ecstasy remains the recreational drug of choice for Australian [15], American [31], and Israeli youths [32]. Amphetamine and amphetamine-type stimulant use is a prevalent problem in Australia [11], the USA [31], and the United Kingdom [3]. According to the UNODC, although ecstasy use in North America and Europe has declined from 2006 to 2017, consumption of ecstasy and cocaine remains high in Australia and New Zealand [3]. The National Drug Household Survey (NDHS) from the AIHW reported a reduction in the recent use of ecstasy between 2010 and 2016 but found an overall increase in the use of cocaine [36]. Globally, the trafficking of stimulants such as cocaine and ecstasy can be considered small in 2015; however, it was noted to be growing rapidly [3].

The NDHS report noted a reduction in methamphetamine usage between 2013 and 2016 [37]. However, this is in contrast to the UNODC World Drug Report, wherein the use of methamphetamine was reported to be increasing in many sub-regions, including North America, Oceania, and most parts of Asia [3].

Limitations

There were several limitations to this literature review. This review only utilized peer-reviewed studies published in English where the full text was available, which may have excluded some literature. Second, although medical records were assumed to be valid and reliable for quality appraisal, there are inherent issues in using medical records for research [6]. Data were not recorded for research purposes, and there is an assumption that all data are recorded accurately. However, there is a spectrum of data quality, especially when using free-text or subjective data. Third, most studies involved a small number of participating hospital EDs or a single ED in a single state, limiting the generalizability of the findings to other jurisdictions and populations.

## Conclusions

The results of this literature review demonstrate that stimulant use contributes a significant challenge to Australian emergency services and healthcare systems. Caring for patients who require mental health care because of stimulant use can affect the safety and well-being of patients and staff in the ED. It can also place greater demand on ED resources. The ED presentations of patients who use stimulants have been documented to be resource-intensive as patients may appear with various physical, behavioral, and mental health conditions that can sometimes require immediate attention and may involve police and ambulance services. Further research is needed to understand better the impact that ED presentations of people who use stimulants impose on the frontline health professionals attending to their care.
